# The Introduction of Robotics to an Outpatient Dispensing and Medication Management Process in Saudi Arabia: Retrospective Review of a Pharmacy-led Multidisciplinary Six Sigma Performance Improvement Project

**DOI:** 10.2196/37905

**Published:** 2022-10-11

**Authors:** Manal Al Nemari, James Waterson

**Affiliations:** 1 Pharmacy Informatics and Automation King Fahad Medical City General Administration of Pharmaceutical Care, Ministry of Health Riyadh Saudi Arabia; 2 Medication Management Solutions Medical Affairs Becton Dickinson Dubai United Arab Emirates

**Keywords:** inventory waste, mislabeling events, no-show returns, inventory stock levels, staff education, task realignment, outpatient, Six Sigma, medication management, medication adherence, risk, pharmacy, health care professional, dispensing, robotics, automation, pharmaceuticals, inventory

## Abstract

**Background:**

Outpatient pharmacy management aims for improved patient safety, improved quality of service, and cost reduction. The Six Sigma method improves quality by eliminating variability, with the goal of a nearly error-free process. Automation of pharmacy tasks potentially offers greater efficiency and safety.

**Objective:**

The goal was to measure the impact that integration of automation made to service, safety and efficiency, staff reallocation and reorientation, and workflow in the outpatient pharmacy department. The Six Sigma problem definition to be resolved was as follows: The current system of outpatient dispensing denies quality to patients in terms of waiting time and contact time with pharmacy professionals, incorporates risks to the patient in terms of mislabeling of medications and the incomplete dispensing of prescriptions, and is potentially wasteful in terms of time and resources.

**Methods:**

We described the process of introducing automation to a large outpatient pharmacy department in a university hospital. The Six Sigma approach was used as it focuses on continuous improvement and also produces a road map that integrates tracking and monitoring into its process. A review of activity in the outpatient department focused on non-value-added (NVA) pharmacist tasks, improving the patient experience and patient safety. Metrics to measure the impact of change were established, and a process map analysis with turnaround times (TATs) for each stage of service was created. Discrete events were selected for correction, improvement, or mitigation. From the review, the team selected key outcome metrics, including storage, picking and delivery dispensing rates, patient and prescription load per day, average packs and lines per prescription, and lines held. Our goal was total automation of stock management. We deployed 2 robotic dispensing units to feed 9 dispensing desks. The automated units were integrated with hospital information technology (HIT) that supports appointments, medication records, and prescriptions.

**Results:**

Postautomation, the total patient time in the department, including the time interacting with the pharmacist for medication education and counseling, dropped from 17.093 to 11.812 digital minutes, with an appreciable increase in patient-pharmacist time. The percentage of incomplete prescriptions dispensed versus orders decreased from 3.0% to 1.83%. The dispensing error rate dropped from 1.00% to 0.24%. Assessed via a “basket” of medications, wastage cost was reduced by 83.9%. During implementation, it was found that NVA tasks that were replaced by automated processes were responsible for an extensive loss of pharmacist time. The productivity ratio postautomation was 1.26.

**Conclusions:**

The Six Sigma methodology allowed for rapid transformation of the medication management process. The risk priority numbers (RPNs) for the “wrong patient-wrong medication error” reduced by a ratio of 5.25:1 and for “patient leaves unit with inadequate counseling” postautomation by 2.5:1. Automation allowed for ring-fencing of patient-pharmacist time. This time needs to be structured for optimal effectiveness.

## Introduction

### Background

Outpatient care and outpatient pharmacy management should aim for improved patient safety, improved quality of service, and reduction in costs [1-3]. The Six Sigma method shares these same goals and aims to improve quality and reduce costs by eliminating variability, with the goal of a nearly error-free process [4]. Automation of non-value-added (NVA) pharmacy tasks that may be undertaken with greater efficiency and more safely by automated units have been identified in the literature [5], and although they will inevitably differ by facility, these are generally given as:

- Stock inputting [5]
- Inventory checks and securing of stock [5]
- Stock rotation by expiry date [6]
- Locating and picking stock [1]
- Guardianship and mandated shift-end counts for controlled substances and high-value medications [7]

King Fahad Medical City began operations in 1984, and the facility has 1200 inpatient beds and receives 500,000 outpatient visits yearly, with an increase of ~15% per year over the study period. It serves the heart of metropolitan Riyadh. Given this large and increasing workload and the increasing complexity of patient conditions being treated in the outpatient department, a systematic review of activity in the outpatient department was undertaken in 2017 as a first step in a change management process aimed at reducing to a minimum NVA pharmacist tasks, improving the patient experience, and improving patient safety. Identified issues at this point were chiefly related to the difficulties in ensuring safe dispensing due to a lack of transparency in the system and the risk of mislabeling products for dispensing, the risk of reinforcing error through labeling the product early in the medication-dispensing chain, pharmacists’ value being lost in NVA tasks, the slow speed of medication picking with rising service requirements per year, a lack of integration between prescribing and dispensing requiring manual checks during picking, and substantial issues over medication availability and avoidable waste through stock expiry.

**Figure 1:**
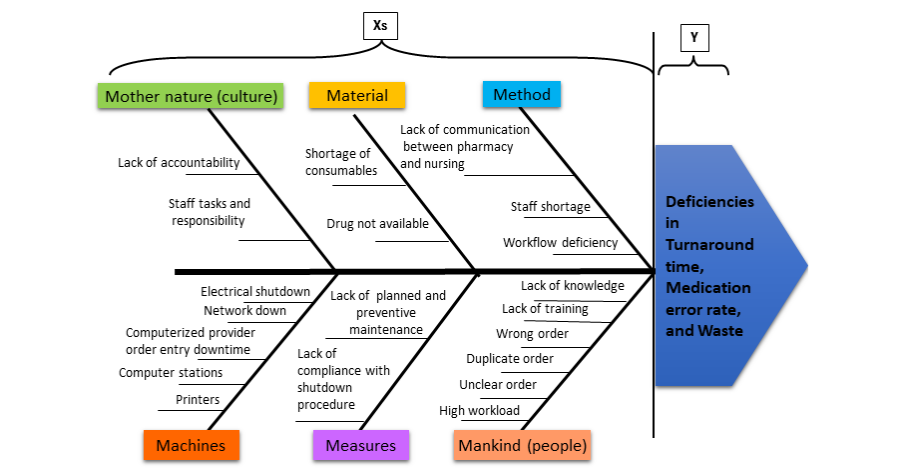
Ishikawa fishbone diagram: input and output deficiencies.

These issues were taken forward to the fuller review and building of an Ishikawa fishbone diagram of input and output deficiencies during the Six Sigma project (see Figure 1).

Our general philosophy during the review and going forward is that the patient experience and patient safety extend beyond metrics, such as waiting time and correct dispensing, and into the growing expectation for pharmacists to deliver effective patient education and counseling [8] and to monitor prescriptions to avoid the risk of patients receiving overwhelming or inappropriate polypharmacy [9,10].

Six Sigma is a methodology that focuses on eliminating the defects in a service or process. It is statistics based and data driven and focuses on continuous improvement [11]. In this respect, it is an excellent choice for services such as ours that have no fixed endpoint but that are rather cycles of service, quality monitoring, and improvement. Another advantage of using Six Sigma in a large and complex organization such as ours is that it produces a road map that improves tracking and monitoring as an integral part of its process.

The define-measure-analyze-improve-control (DMAIC) process is the central “spine” of the Six Sigma process. For health care, the fundamental processes first introduced into the manufacturing industry in the 1980s have been adapted to emphasize the preventive component of error reduction as this makes the process fit with other fundamental risk management processes, such as failure mode effect analysis and root-cause analysis of near misses and actual incidents [4]. The fundamentals of DMAIC for health care projects are as follows:

- Define: Delineate in detail internal customers and external customers (patients and family) and what each customer type wants and needs. Define the processes in use and their capabilities and clearly describe objectives for projected improvement efforts [12].
- Measure: Delineate the quality characteristics that would reflect improvement in customer satisfaction and process performance. Measurement also involves the creation of metrics on which the improvement efforts will be targeted.
- Analyze: Analyze data using analytical tools, such as Pareto analysis, process flow diagrams, fishbone diagrams, and statistical process control charts, to identify necessary design and process modifications for achieving customer satisfaction and performance objectives.
- Improve: Allocate resources and ring-fence them so that design and process modifications needed for improvement can be implemented rapidly and comprehensively [13].
- Control: Monitor continuously using quality management tools to ensure that the performance improvements are maintained.

### Objective

The overall goal of applying a Six Sigma process improvement project plan and the objective of this study is to improve and to measure any improvement that automation, staff reallocation and reorientation, workflow change, and integration would make to service, safety, and efficiency in an outpatient pharmacy department.

**Figure 2:**
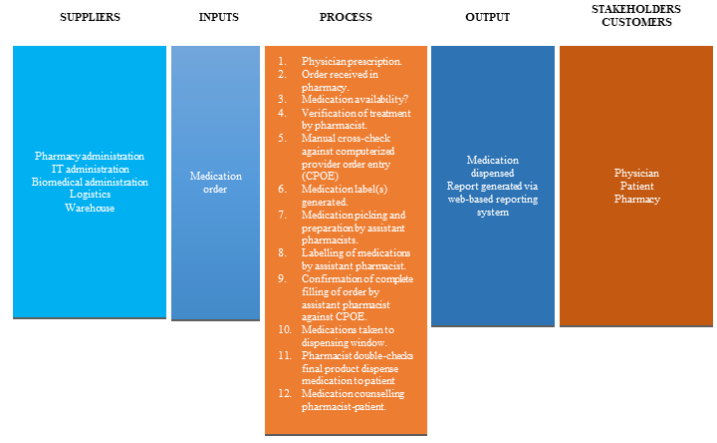
SIPOC end-to-end mapping of medication to patient actions. CPOE: computerized provider order entry; SIPOC: supplies, inputs, process, outputs, customers.

The DMAIC Six Sigma change management process works most effectively when a problem statement is created to focus on an area of concern, a condition to be improved upon, or a difficulty to be eliminated [14]. We undertook a series of analyses to define the characteristics of our problem and its individual issues and to generate our problem definition:

- Supplies, inputs, process, outputs, customers (SIPOC) end-to-end actions overview (see Figure 2). The value of SIPOC analysis is that it identifies chokepoints at each stage of the input and output processes. The breakdown should have the least number of steps feasible to adequately describe the total process and ends with the internal and external stakeholders to ensure that all change is directed at improving results directly related to these individuals. In this study’s SIPOC, the bulk of change was in the third stage of the process, and this directly impacted the final output in terms of medication turnaround time (TAT) and patient waiting time, ring-fenced time for counseling, and medication safety, as indicated by the fourth output stage for reported incident rates.
- Critical steps within SIPOC (see Table 1). As discussed earlier, the bulk of the activity was at the third stage of SIPOC. A more detailed process mapping of the critical steps with the average TAT and types of tasks (value-added [VA] or NVA) was created to find potential delay points and opportunities for change.
- Customer and stakeholder segmentation and identification (see Textbox 1).

**Table 1:** Outpatient medication-dispensing process map before Six Sigma process application, with critical steps’ mapping and TATs^a^.

Step	Procedure	Projected TAT (digital minutes)	Stakeholder	VA^b^/NVA^c^	Internal/external failure	Control/inspection
1	Order a medication.	0.1	Physician	N/A^d^	Unclear order Physician unavailable	Outpatient clinic
2	Receive the order.	0.2	Pharmacist	VA	Unavailability of staff Unclear order	Support from other staff or supervisor; call doctor
3	Verify the order.	1.0	Pharmacist	VA	Unavailability of medication	Substitute/borrow
4	Enter the medication into the CPOE^e^.	1.0	Pharmacist	VA	Unavailability of reference to check	Hardcopy reference available
5	Process the medication though the CPOE.	1.0	Pharmacist	VA	Network downtime	Pharmacy manual backup system
6	Generate a medication label.	0.3	Pharmacist	NVA	Network downtime	Pharmacy manual backup system
7	Pick the medication.	8.0	Pharmacist	NVA	Unavailability of staff	Support from other staff or supervisor
8	Label the medication.	0.5	Pharmacist	NVA	Lost label	Reprint label
9	Check the completeness of the order.	1.0	Pharmacist	NVA	Unavailability of staff	Use technicians
10	Transport the medication to the dispensing window.	1.0	Pharmacist	NVA	Unavailability of staff	Use technicians
11	Double-check the dispensed medication against the prescription.	1.0	Pharmacist	VA	Unavailability of reference to check	Hardcopy reference available
12	Dispense the medication.	2.0	Pharmacist	VA	Unavailability of staff Refused by patient	Support from other staff or supervisor; call doctor
13	Counsel the patient and check back patient understanding.	1.0	Pharmacist	VA	Unavailability of staff Refused by patient	Support from other staff or supervisor; call doctor
14	The medication is received and instructions understood.	1.0	Patient	N/A	Unavailability of staff Refused by patient	Support from other staff or supervisor; call doctor

^a^TAT: turnaround time.

^b^VA: value-added (total projected TAT=7.2/19.1, 38%, digital minutes; number of VA tasks=7/14, 50%).

^c^NVA: non-value-added (total projected TAT=10.8/19.1, 57%, digital minutes; number of NVA tasks=5/14, 36%).

^d^N/A: not applicable (total projected TAT=1.1/19.1, 5%, digital minutes; number of N/A tasks=2/14, 14%).

^e^CPOE: computerized provider order entry.

**Identified customer and stakeholder segments by priority.**

**Internal**

- Pharmacists
- Technicians
- Hospital information technology (HIT) team
- Physicians
- Warehouse stock team

**External**

- Patients
- Pharmaceutical vendors
- Technology vendors

The end-to-end survey of the process showed weaknesses at multiple points in terms of safety, quality, and efficiency. The survey identified the following areas of concern: medication-picking times, labeling accuracy and times, availability of medications, wastage through expiry and patient no-show, TAT for reintroduction of unused medications, and time taken in completing inventory. Of particular concern was the difficulty in constructing a viable risk management mitigation plan based on failure mode effect analysis (FMEA) that was undertaken concurrently due to a lack of transparency in the workflow and a lack of data generated by the system.

In classic FMEA planning [15] for any high-risk activity, and particularly activities with a high risk of low chance or no chance of detection of errors, the activity is broken down into individual steps, each of which can mitigate, correct, or annul any error in the previous steps. Our SIPOC did indicate a logical FMEA process to a certain degree, as steps involving the risk of error in terms of cross-checks versus the computerized provider order entry (CPOE) and picking preceded the final verification checks before the ultimate interaction with the patient by the pharmacist. However, previous steps, in particular the labeling and transport of the medication to the dispensing window by a pharmacist, were identified as adding to the risk of reinforcement of error during the final pharmacist check before release of the medication to the patient. For example, the risk during medication picking of a look-alike sound-alike (LASA) error. LASA error rates have been reported as being as high as 25.9% of all reported medication errors [16]. Furthermore, the manual application of a patient label at this stage risk influencing the pharmacist during the final check and may in fact risk reinforcing the chance of error rather than averting it [17,18].

It was also noted during our review that the time spent with the patient for medication counseling was potentially too short to allow for optimal counseling and education [19]. The patient-pharmacist time was also likely to be “squeezed” by the need to manage patient volume and to handle increasing transaction rates per day [20].

We wanted our medication chain to have improved detection, and decreased risk, of error at each stage of the medication chain. FMEA risk priority numbers (RPNs) were calculated for all perceived risks in day-to-day operations of the outpatient pharmacy department. In this study, we focused on the 2 conditions laid out earlier, as they are central to patient safety but also reflect possible efficiency gains that could be obtained by introducing automation. The FMEA RPNs (severity score [SS] × probability score [PS] × detectability score [DS]) were, respectively:

Misinterpretation of prescription-wrong patient-wrong medication error:

SS × PS × DS = 6 × 3 × 7 = 126

Patient leaves unit with inadequate counseling:

SS × PS × DS = 4 × 5 × 3 = 60

The high PS for inadequate counseling was based on the degree of variability in patient service time, discussed later. Our scores overall were in line with risk calculations made in other centers reviewing manual dispensing systems for outpatient departments [3]. See Multimedia Appendix 1 for a fuller description of the process and improvement criteria according to the Institute for Healthcare Improvement [21].

These analyses allowed for a Six Sigma problem statement to be generated:

The current system of outpatient dispensing denies quality to patients in terms of waiting time and contact time with pharmacy professionals, incorporates risks to the patient in terms of mislabeling of medications and incomplete dispensing of prescriptions, and is potentially wasteful in terms of time and resources.

## Methods

### Study Design

The study lasted 20 months (April 2019-December 2020), with a go-live for the automated pharmacy after 3 months (July 2019).

To assess the impact of changes brought about by automation, task reassignment, and workflow restructuring, it was necessary to establish further metrics; we reviewed our Six Sigma change process and reviewed the steps delineated in Table 1 and its process map analysis of discrete events with an average TAT for each step, with a risk of delay by cause for each stage (Table 2). Going forward, the TAT was calculated from multiple observations at each step of the process.

It was possible to directly review causes for protracted TATs and frequent delays. Given the substantial number of stakeholders and multiple data points, an Ishikawa fishbone cause-and-effect diagram was built to assist the team of internal stakeholders in brainstorming to capture the sources of process variation, to drill down for the causes of delay, to investigate each detrimental effect, and to determine correctable and improvable causes [22].

The discrete events identified were selected for correction, improvement, or mitigation. The final task during our appraisal was to return to the TAT and delay assessment and, in conjunction with the Ishikawa exercise, to decide on the final variables and derived metrics that would indicate, most accurately, reliably, and appropriately, the effect of any changes we put in place. The team, supported by the facility’s analytics department, selected 11 key measurable outcome variables and derived metrics (see Table 3). The review also aided with team selection as we identified process variations and chokepoints hindering improvement (see Figure 1) and could recruit personnel directly involved at these points into the project team.

**Table 2:** Process map analysis for average TAT^a^ and delay postautomation.

Step	Procedure	Projected TAT (digital minutes)	Stakeholder	VA^b^/NVA^c^	Internal/external failure	Control/inspection
1	Order a medication.	0.1	Physician	N/A^d^	Unclear order Physician unavailable	Outpatient clinic
2	Receive the order.	0.2	Pharmacist	VA	Unavailability of staff Unclear order	Support from other staff or supervisor; call doctor
3	Verify the order.	1.0	Pharmacist	VA	Unavailability of medication	Substitute/borrow
4	Enter the medication into the CPOE^e^.	0.1	Integration	N/A	Unavailability of reference to check	Hardcopy reference available
5	Process the medication though the CPOE.	0.1	Integration	N/A	Network downtime	Pharmacy manual backup system
6	Generate a medication label.	0.1	Automation/integration	N/A	Network downtime	Pharmacy manual backup system
7	Pick the medication.	1.0	Automation/integration	N/A	Unavailability of staff	Support from other staff or supervisor
8	Label the medication.	0.1	Automation/integration	N/A	Lost label	Reprint label
9	Check the completeness of the order.	0.1	Automation/integration	N/A	Unavailability of staff	Use technicians
10	Transport the medication to the dispensing window.	0.2	Automation/integration	N/A	Unavailability of staff	Use technicians
11	Double-check the dispensed medication against the prescription.	1.0	Pharmacist	VA	Unavailability of reference to check	Hardcopy reference available
12	Dispense the medication.	1.8	Pharmacist	VA	Unavailability of staff Refused by patient	Support from other staff or supervisor; call doctor
13	Counsel the patient and check back patient understanding.	5.0	Pharmacist	VA	Unavailability of staff Refused by patient	Support from other staff or supervisor; call doctor
14	The medication is received and instructions understood.	1.0	Patient	N/A	Unavailability of staff Refused by patient	Support from other staff or supervisor; call doctor

^a^TAT: turnaround time.

^b^VA: value-added (total projected TAT=9.0/11.8, 76%, digital minutes; number of VA tasks=5/14, 56%).

^c^NVA: non-value-added.

^d^N/A: not applicable (total projected TAT=2.8/11.8, 24%, digital minutes; number of N/A tasks=9/14, 44%).

^e^CPOE: computerized provider order entry.

**Table 3:** Key measurable outcome indicators.

Variable and derived metric	Type of metric	Assessment methods	Pre-reengineering metrics	Postreengineering metrics	Inferred metric
Patient waiting time from presentation to departure with correct medications and appropriate counseling	Outcome indicator	Total time in department – time interacting with pharmacist for medication education and counseling	17.093	11.812	Improvement in adherence, reduction in adverse drug events (ADEs) at home
Completeness of all dispensed prescriptions	Outcome indicator	Percentage of complete prescriptions dispensed versus orders Error rate per 1000 items dispensed	Data of 6 months at initial and immediate stages of automation takeover (using automated pharmacy systems to account for lack of data prerobotic pharmacy) Mean 3.0% (SD 5.07%) Orders sampled (N=14,991)	Data of 6 months following go-live (1) and 6 months of system stabilization (2) 1: mean 2.99% (SD 2.82%) 2: mean 1.83% (SD 0.99%)	Pre-post availability of medications in percentage inventory transparency
Accuracy of all dispensed medications	Outcome indicator	Medication error reporting via incident reporting system (one year pre- and post-engineering via web-based incident reporting system) In-out discrepancies/day Number of mislabeling events caught Data of 1 year pre- and postreengineering	Mean 1.00% (SD 0.279%)	Mean 0.24% (SD 0.299%)	Workflow FMEA^a^ capacity to prevent error reaching patient
FMEA RPN^b^ for “misinterpretation of prescription-wrong patient-wrong medication error” reduced from the initial score of 126	Outcome indicator	Recalculate FMEA RPN postreengineering	SS^c^ × PS^d^ × DS^e^ = 6 × 3 × 7 = 126	SS × PS × DS = 6 × 1 × 4 = 24 PS reduced by a ratio of 4:1 (1.0%:0.24%) DS reduced as more complete information and transparency of workflow increased	Safety of process
FMEA RPN for “patient leaves unit with inadequate counseling” reduced from the initial score of 60	Outcome indicator	Recalculate FMEA RPN postreengineering Pharmacist spends more time for consultation in digital minutes or % per interaction	SS × PS × DS = 4 × 5 × 3 = 60	SS × PS × DS = 4 × 2 × 3 = 24 5 digital minutes of time counseling the patient ring-fenced compared to 1 digital minute in preautomation; however, time still being structured for optimal effectiveness	Reduced likelihood of unscheduled return to hospital caused by medication nonadherence
Pharmacist deployment to VA^f^ vs NVA^g^ tasks	Outcome indicator	NVA tasks by employed digital minutes per item dispensed × average number of items/year dispensed Time not including maintenance tasks and manual inventory	Total 17.093 digital minutes per prescription VA=6.44 digital minutes per item NVA=9.67 digital minutes per prescription N/A^h^=0.98 digital minutes per prescription NVA time=mean 28,831 (SD 2992.60) new prescriptions/month × 9.67 × 12=3,345,549.2 digital minutes=2323 days 7 hours/year (24-hour service) 1 FTE^i^=225 days/year=10.33 FTEs	Total 11.812 digital minutes per item NVA=0 VA=10.247 digital minutes per item N/A=1.565 digital minutes per item NVA time=0	Ability to apply human resources to VA, patient-centered activities
Prescriptions filled	Process quality indicator	Items/day/year	Data of 1 year pre-reengineering≈7824 items/day≈2,856,000 items/year	Data of 1 year postreengineering≈9884 items/day≈3,607,400 items/year	Fluctuation management, pharmacist productivity (productivity ratio=1.26)
Waste through expiry, loss, or failure to reintroduce into the system	Outcome indicator	Calculated by cost of a “basket” of 8 diverse prescriptions Percentage waste change postreengineering (Table 4)	US $124,592.24 lost to waste (Table 4)	US $20,017.98 lost to waste 83.9% reduction in waste across basket (Table 4)	Reduction of expired stock loss: more efficient, leaner stock (lower stock level, lower cash binding)
Staff education on automated processes and workflow changes, and direction on use of freed-up time: polypharmacy, counseling techniques	Process quality indicator	Directed in-service education time/month	In-service teaching time devoted to workflow per month=mean 825 (SD 375) digital minutes	In-service teaching time devoted to use of freed-up time per month=mean 637.5 (SD 341.64) digital minutes	Move pharmacists from functional roles to innovative roles

^a^FMEA: failure mode effect analysis.

^b^RPN: risk priority number.

^c^SS: severity score.

^d^PS: probability score.

^e^DS: detectability score.

^f^VA: value-added.

^g^NVA: non-value-added.

^h^N/A: not applicable.

^i^FTE: full-time employee.

**Table 4:** “Basket” of 8 prescription medications reviewed for costs of waste by expiry or incorrect storage over 1 year.

Medication name and dose	Unit value (US $)	Unit waste pre-reengineering	Cost waste pre-reengineering (US $)^a^	Unit waste postreengineering	Cost waste postreengineering (US $)^b^
Everolimus 10 mg	151.88	630	95,681.25	90	13,668.75
Isoniazid 10 mg/mL	65.41	9	588.67	5	327.04
Lipase 5000 international units (IU) + amylase 3600 IU + protease 200 IU granules	59.40	215	12,771.00	30	1782.00
Apomorphine 10 mg/mL	45.36	300	13,608.00	85	3855.60
Cetrorelix 0.25 mg injection	33.40	9	300.62	5	167.01
Tacrolimus 0.03% cream	14.85	55	816.75	6	89.10
Methotrexate 7.5 mg/0.15 mL syringe	12.96	34	440.64	3	38.88
Cefdinir 125 mg/5 mL suspension	8.96	43	385.31	10	89.61

^a^Total cost waste pre-reengineering=US $124,592.24.

^b^Total cost waste postreengineering=US $20,017.99.

The rationale for the selection of variables was that they directly address the key areas of the problems and questions we were trying to solve, as identified in our Six Sigma process mapping and Ishikawa deconstruction, and they also work across the spectrum of a complex of issues that are interrelated and interdependent: this was a fundamental reason for selecting Six Sigma as our change methodology, as improvements in 1 variable could impact multiple metrics and the interplay between them is well described through Six Sigma with its identification of chokepoints to improvement. The variables identified for metric development were the risk of dispensing errors, the risk of the system reinforcing the error risk rather than mitigating it, the question of how much transparency the system has, the loss of staff value, the patient experience, maintaining adequate throughput in the system to allow for ring-fenced pharmacist-patient time, ensuring medication availability through integration, and reducing medication loss through expiry/misplacing. The purpose of the derived metrics was that they triangulate with data the variables we wanted to investigate, which are difficult to address directly (Table 5).

**Table 5:** Selected variables and derived metrics with rationales.

Variables	Derived metrics addressing variables	Selection rationale for variables and derived metrics
Patient experience, ring-fenced pharmacist-patient time, staff value and use	Patient waiting time from presentation to departure with correct medications and appropriate counseling	Indirect measurement of patient satisfaction and experience (linked to waiting time [1]), indirect indication of probability of ring-fenced pharmacist-patient time being maintained
Risk of dispensing error, dispensing throughput, medication loss, system transparency	Completeness of all dispensed prescriptions	Direct measurement of complete orders, indirect assessment via incident reporting and root-cause analysis of medication not being available
Risk of dispensing error, system transparency	Accuracy of all dispensed medications	Direct measurement of error rate via an incident reporting system, FMEA^a^ indirect assessment of error detectability via FMEA scoring pre- and postautomation
Risk of dispensing error, system transparency	FMEA RPN^b^ for “misinterpretation of prescription-wrong patient-wrong medication error” reduced from the initial score of 126	Change in RPN calculated from PS^c^ and DS^d^
Ring-fenced pharmacist-patient time, system transparency, staff value and use	FMEA RPN for “patient leaves unit with inadequate counseling” reduced from the initial score of 60	Change in RPN calculated from PS and DS
Ring-fenced pharmacist-patient time, system transparency, staff value and use	Pharmacist deployment to VA^e^ vs NVA^f^ tasks	Semidirect calculation derived from calculation of time for NVA tasks eliminated by automation against total unit throughput in medications dispensed
Dispensing throughput, system transparency, staff value and use	Prescriptions filled	Direct measurement of ability to meet demand and ability to measure volume handled, indirect measurement of how successfully automation and human systems are interacting
Medication loss	Waste through expiry, loss, or failure to reintroduce into the system	Direct and frequent inventory of medication stock and waste, expired medications counted, derived calculation of misplaced medications
Ring-fenced pharmacist-patient time, patient experience, staff value and use	Staff education on automated processes and workflow changes, and direction on use of freed-up time: polypharmacy, counseling techniques	Indirect indication of staff engaging in counseling and in patient engagement

^a^FMEA: failure mode effect analysis.

^b^RPN: risk priority number.

^c^PS: probability score.

^d^DS: detectability score.

^e^VA: value-added.

^f^NVA: non-value-added.

Our review of the materials and solutions needed for the automated “heart” of our reengineering of the process and management of the outpatient pharmacy was guided by a review of the literature [3,5]. Technology selection in terms of required storage, picking, and delivery rates was made through a review of 2017-2019 pack-dispensing rates (mean 292,662, SD 34,301 packs/month; mean 10,452, SD 1225 packs/day) for a mean patient load of 42,663 (SD 2992) patients/month, or 1524 (SD 107) patients/day.

As noted before, we experienced an increase of ~15% patients/year using the service, and we planned for this required extra capacity.

The outpatient pharmacy operated, both pre- and postautomation, a 24-hour service, with peak times from 9:00 a.m. to noon and from 4:00 p.m. to 6:00 p.m. This was important to note for planning as no restocking could be expected to take place during these periods, with both robotic units dedicated to meet the dispensing demand.

Our goal was to achieve as extensive an automation of the processes of stock management as possible; therefore, we investigated systems with semi- and fully automatic input, and this was planned to take place during low-patient-volume hours at a minimum rate of 350-500 packs/hour input.

BD Rowa Vmax 160 hardware was selected based on the aforementioned criteria for picking and input speed and positive integration attributes. Two machines were purchased, each with dimensions of 7.4 m length × 1.6 m width × 2.9 m height. Each unit has a capacity for ~12,500 medications, with a potential high-density storage capacity of 18,300-20,100 medications. The external architecture serves 9 dispensing desks via spiral chutes, fed by 2 unidirectional belts with feed gates, serviced by 1 bidirectional belt feeding from 4 exit points of the 2 robot picking units. Given the substantial volumes of medications dispensed per day in our outpatient department, and to achieve the higher rate of medication picking and dispensing that we required to meet our targets, we opted not to connect the 2 units but instead to stock both units fully with required medications. Connecting the units has some advantages in that if 1 robotic unit is operating at a faster rate for picking, it can take over a greater load and dispense more of the served dispensing desks. However, this crossover of activity between units can interrupt picking and create small, but important, breaks in dispensing activity.

Refill of the robotic units was via an electronic refill system that triggers stock requests versus par levels for each medication held against a continuous inventory and consumption check.

The project took an open approach to integration with existing hospital information technology (HIT), as planning was in place for replacement of the information environment of 2017 with an organization-wide conversion to an Epic Systems Corporation electronic medical record (EMR) with integration into the EMR of all services and health information and EMR support for appointments, medication records, and prescription by 2022.

FutureGate Pharmaflow architecture was chosen for initial and ongoing integration of the robotic pharmacy. This solution can interface via Health Level Seven (HL7) and is therefore flexible enough to operate through a changing HIT environment.

Where extra notation and extra labeling at medication input were required for picking by the robotic pharmacy, these barcodes were generated from the Pharmaflow formulary manager, although the gradual introduction of the Global Trade Item Number (GTIN) for medications over the go-live and study period reduced this need somewhat. Whole-box dispensing was used.

The Pharmaflow solution was also chosen based on ensuring continuity of service. The solution’s VMware that supports the robotic pharmacy’s interoperability is entirely within the facility’s firewall, and there are no requirements for inbound ports to be opened. Urgent maintenance requests are managed by a remote access broker via a Secure Sockets Layer (SSL) over Transmission Control Protocol (TCP) port 443 (outbound rule only). Virtual proxy network (VPN) access is initiated by our facility if access by vendor engineers is required for remote server maintenance. The user identification and the date and time of any action are electronically logged.

The question of wastage of medications and whether this reduces following the implementation of automation was addressed by a retrospective review of manual inventories for loss through expiry or incorrect storage of a basket of 8 diverse medications pre-reengineering and a review of automated reports postreengineering for these same medications (see Table 4). The medications were selected to be representative of a wide selection of the types of medication we carry in our outpatient inventory, from slow-moving items with small-inventory-volume items to commonly used medications with larger holding stock volumes, specialist items, and parenteral and enteral products. Higher-cost items were not used, as their single-item value could skew the inventory costs savings and they were subject to individual stock control measures.

This targeted approach was taken as data in the prephase, due to the difficulties in undertaking a systemwide inventory, were limited.

### Ethical Considerations

As data are continually collected from medical devices across our facility, and all nursing and medical staff are aware of ongoing collection and analysis of actual and near-miss events, no formal consent was required from the ethics committee of King Fahad Medical City for data gathering.

### Study Procedure

The data recorded for analysis were patient-anonymized for hospital number, gender, name, date of birth, or other identifiable material. All employees active in the outpatient unit were informed of the purpose of the study and the data collection taking place. Furthermore, the change management process and attendant data gathering are a consistent part of the facility’s process of Joint Commission International (JCI) quality improvement and zero-harm targets. According to facility protocols, the pharmacy department owns all medication management automation data and is recognized as the lead department for medication safety. BD Clinical and FutureGate Global Customer Services were engaged to optimize the automated solution. BD Medical Affairs was requested to undertake a deeper analysis of the data. The medical affairs department of BD operates as a distinct arm outside of the commercial operations of the company.

#### Inclusion Criteria

All formulary tablet and capsule orders were dispensed via the outpatient pharmacy as original-pack medications either as newly prescribed or as represcribed items; this included whole-box brand name medications entered into the system via manufacturers’ barcode identification and whole-box generic medications entered into the system via facility-applied barcode identifiers. Ready-for-use suspensions and suspensions requiring reconstitution were included, as were enzyme supplement packs in granular form. Prefilled medication syringes and powder and solvent injection kits were included as packs and individual items according to prescriptions.

#### Exclusion Criteria

The study did not include unit-dose medications for suspensions, tablets, or capsules. Patient-named medication and blister packs were excluded.

## Results

Summary weekly reports from preautomation showed mean and median service times slightly below the projected TAT of 19.1 digital minutes for 75% of patients; however, this was ~47 digital minutes for 95% of patients. Postautomation results from early 2020 showed that the mean average TAT fell to 11.8 digital minutes, with 88% of patients served within 20 digital minutes (see Table 6).

**Table 6:** Sample prescription dispensing TATs^a^ pre- and 3 months postautomation.

Sample	Preautomation	3 months postautomation
Mean (SD)	17.093 (5.743)	11.812 (3.821)
Variance	32.977	14.597
Skewness	1.26864	0.840425
Kurtosis	2.21838	0.271411
N	53	53
Minimum	8.128	6.456
1st quartile	13.619	9.026
Median	15.829	11.096
3rd quartile	20.097	14.029
Maximum	36.635	21.783
95% CI for mean (SD)	15.510-18.676 (4.280-7.105)	10.759-12.865 (3.207-4.727)
95% CI for median	14.702-16.987	9.599-12.637

^a^TAT: turnaround time.

## Discussion

### Principal Findings

The Six Sigma methodology allowed for rapid transformation of the medication management process. The RPN for “wrong patient-wrong medication error” reduced by a ratio of 5.25:1 and for “patient leaves unit with inadequate counseling” postautomation by 2.5:1. As per the Institute for Healthcare Improvement guidelines [21], a 50% (2:1) reduction in the RPN indicates a successful FMEA process. The percentage of incomplete prescriptions dispensed versus orders decreasing from 3.0% to 1.83% is in line with previous studies of risk analysis [3]. The dispensing error rate drop from 1.00% to 0.24% is perhaps a reflection of the change in workflow with a reduction in the risk of dispensing from “upstream” labeling errors in the pre-reengineering process.

The FMEA detectability scores are a continuing concern for counseling, although automation has allowed for ring-fencing of patient-pharmacist time. The difficulty is, of course, quantifying the effectiveness of counseling. A structured approach to patient education and the use of the “teach back” methodology may allow us to quantify the effectiveness of patient counseling more completely. It is notable that the 95% CI for the median time for medication dispensing carries a narrower dispersal of times (9.599-12.637 vs 14.702-16.987), and this reduction in variability, as well as the reduced time overall, gives us more confidence for planning for consistent structured pharmacist-patient counseling time. The extra time has been created from a reduction in NVA pharmacist tasks and reduced patient waiting time.

The evolving EMR project may be of assistance in assessing the impact of this increased patient-pharmacist time, as it can help us determine the volume of admissions related to nonadherence to medication or incorrect medication usage at home by patients. Small-scale studies have indicated that in Saudi Arabia, hospital admissions due to medication errors at home account for 14.7% of emergency room admissions, with failure to take or receive medication being responsible for 47.2% of these presentations [23].

Personnel were a central component of the Ishikawa diagram of deficiencies, and it is notable how much NVA task time was uncovered by the change to automation in terms of full-time employee (FTE) time devoted to these types of tasks. The substantial FTE time saved will not lead to a reduction in workforce; redeployment and reorientation of staff through education for more patient-directed activity are ongoing, and given that we expect ongoing increases of ~15% patients/year using the service, we recognize the need for extra capacity in all areas of the department.

We undertook a series of analyses to define the characteristics of our problem, and 1 major problem we encountered was that many of the manual activities we undertook preautomation generated no data or data that were hard to obtain and appraise. With automation, a great deal of “passive” data collection takes place, giving improved transparency to the system.

For example, the selection of a “basket” of medications for wastage review was required due to a lack of data preautomation, and the cost saving calculated from this was substantial. Postautomation, with a dynamic inventory, we can extend these reviews across the entire stock held within the robotic pharmacy. Other studies of robotic pharmacy installations have shown a return on investment (ROI) within 3.5-3.75 years [1,6], with reduced wastage as a significant component of this return.

### Conclusion

Our data indicate that the efficiency and safety of the system are improving with time. We believe that these ongoing improvements are related to staff having “learned” the technology and becoming increasingly proactive in its use and being able to use the new systems more effectively, as well as exploiting opportunities presented by automation. Our initial Six Sigma problem statement included the issue that our system was “potentially wasteful in terms of time and resources.” A key aspect of the reengineering we undertook is that we can more clearly identify where these potential losses are and more accurately target them.

## Appendix

Multimedia Appendix 1 Summary of Institute for Healthcare Improvement implementation of failure mode effect analysis processes, with selected component processes.

## Abbreviations

CPOE: computerized provider order entry
DMAIC: define-measure-analyze-improve-control
DS: detectability score
EMR: electronic medical record
FMEA: failure mode effect analysis
HIT: hospital information technology
LASA: look-alike sound-alike
NVA: non-value-added
PS: probability score
RPN: risk priority number
SIPOC: supplies, inputs, process, outputs, customers
SS: severity score
TAT: turnaround time
VA: value-added
