# Supplementary material for: The Introduction of Robotics to an Outpatient Dispensing and Medication Management Process in Saudi Arabia: Retrospective Review of a Pharmacy-led Multidisciplinary Six Sigma Performance Improvement Project
Source: JMIR Hum Factors. 2022 Oct 11;9(4):e37905. doi: 10.2196/37905 (PMC9597422; doi:10.2196/37905)
Supplement: Multimedia Appendix 1 [file humanfactors_v9i4e37905_app1.docx]

**Summary of Institute for Healthcare Improvement implementation of FMEA processes, with selected component processes.**

| Steps in the process | Failure mode | Failure causes | Failure effects | Likelihood of occurrence (1-10) | Likelihood of detection (1-10) | Severity (1-10) | Risk profile number | Actions to reduce occurrence of failure |
| --- | --- | --- | --- | --- | --- | --- | --- | --- |
| Dispensing of prescribing medication to patient | Misinterpretation of prescription: Wrong Patient: Wrong Medication Error: | Transcription error, picking error, incorrect labelling, lack of final check, poor patient understanding, lack of detection processes. | Patient admission with ADE, therapeutic failure, treatment costs, waste. | 3 | 7 | 6 | 126 | Integration between CPOE and picking, medication verification steps reordering, teach back process with patient |
| Patient counseling for new and repeat prescriptions. | Patient leaves unit with inadequate counseling. | Variability in patient service time, inadequate staff training, lack of structured process, patient resistance. | Patient admission with ADE, lack of adherence, unrecognized polypharmacy. | 5 | 3 | 4 | 60 | Ring-fencing of time with patient, distinct plan for counseling: Teach back process with patient, triggers for polypharmacy review. |
